# Designing 3D Digital Metamaterial for Elastic Waves: From Elastic Wave Polarizer to Vibration Control

**DOI:** 10.1002/advs.201900401

**Published:** 2019-06-14

**Authors:** Huan Liu, Quan Zhang, Kai Zhang, Gengkai Hu, Huiling Duan

**Affiliations:** ^1^ State Key Laboratory of Turbulence and Complex Systems Department of Mechanics and Engineering Science BIC‐ESAT College of Engineering Peking university Beijing 100871 China; ^2^ School of Aerospace Engineering Beijing Institute of Technology Beijing 100081 China; ^3^ Key Laboratory of Dynamics and Control of Flight Vehicle School of Aerospace Engineering Beijing Institute of Technology Beijing 100081 China; ^4^ CAPT HEDPS and IFSA Collaborative Innovation Center of MoE Peking University Beijing 100871 China

**Keywords:** 3D digital metamaterials, elastic wave polarizers, local resonances, tunable, vibration control

## Abstract

Elastic wave polarizers, which can filter out linearly polarized elastic waves from hybrid elastic waves, remain a challenge since elastic waves contain both transverse and longitudinal natures. Here, a tunable, digital, locally resonant metamaterial inspired by abacus is proposed, which consists of 3D‐printed octahedral frames and built‐in electromagnets. By controlling current in the electromagnets, each unit cell exhibits three digital modes, where the elastic waves have different characteristics of propagation under each mode. A variety of waveguides can be formed by a combination of the three modes and desired polarization can be further filtered out from hybrid elastic waves in a tunable manner. The underlying mechanism of these polarizer‐like characteristics is investigated through a combination of theoretical analysis, numerical simulation, and experimental testing. This study provides a means of filtering out the desired wave from hybrid elastic waves, and offers promise for vibration control of particle distribution and flexible structure.

The polarization of classical waves is ubiquitous throughout nature, including polarized electromagnetic and optical waves, and polarized elastic wave. The study of polarized waves has thrived first in electromagnetism and optics. Many intriguing applications have been successfully explored, such as liquid crystal display (LCD) technology,^1^, ^2^ telecommunication,^3^, ^4^ photoelasticity,^5^ and polariscope.^6^ Elastic wave as another important classical wave, its polarization plays a vital role in geology,^7^ seismology,^8^, ^9^ and engineering.^10^ However, compared with electromagnetic waves and fluid‐borne acoustic waves, elastic waves contain both transverse and longitudinal natures,^11^ which make elastic waves complex to handle. Furthermore, waveform conversion between longitudinal and transverse waves is pervasive during wave propagation. It is therefore more challenging to obtain linearly polarized waves.

Elastic metamaterials^12^, ^13^, ^14^, ^15^, ^16^ with a variety of unusual properties, such as negative effective density,^17^, ^18^ negative effective modulus,^19^, ^20^, ^21^ and double negative,^22^, ^23^ are expected to deal with the complicated polarization features. Remarkably, recent studies based on locally resonant metamaterials^24^ have developed attractive polarization properties of elastic waves, such as fluid‐like behavior^25^, ^26^ and polarized bandgaps.^25^ However, to the best of the authors' knowledge, only one type of polarized wave can be filtered out from hybrid elastic waves in a given frequency.^25^ Moreover, owing to the immutable microstructures, these systems can only be used in a fixed frequency range with unchangeable performance. To overcome these restrictions, elastic metamaterials are required to polarize various hybrid waves in a tunable manner. One Chinese counting tool—abacus, which is composed of multiple strings of beads, can realize different counting results by switching the position of its beads. Inspired by its shape and the way of implementing counting functionality, we propose a 3D tunable, polarizer‐like, elastic metamaterial that contains a 3D‐printed frame and eight built‐in electromagnets. By controlling the current to change the position of the electromagnets, each unit cell can exhibit three modes, and expected polarization of elastic waves can be obtained from hybrid waves in a tunable manner. Our work offers a novel approach to obtain linearly polarized elastic waves, and significant potential applications for vibration control on particle manipulation and flexible structure.

Starting with the proposed metamaterial as shown in **Figure** **1**a, a 3D‐printed octahedral frame is first fabricated. Inside the frame are three circular plates, two cylindrical beams (Young's modulus, *E*_t_ = 2 GPa) at the ends and four rectangular beams (Young's modulus, *E*_c_ = 1 GPa) at the center. Eight electromagnets are then embedded into the frame. Two of the eight electromagnets are adhered to the two sides of the central plate, and one each to the top and the bottom circular plates. The remaining four are allowed to move up and down between the fixed ones. By controlling current of each electromagnet, three symmetric modes—M1, M2, and M3—are generated. To switch modes more conveniently and intelligently for the metamaterial, we established a mode control system. By manipulating the software in this system, the three modes can be switched simply and independently (see details in the Supporting Information).

**Figure 1:**
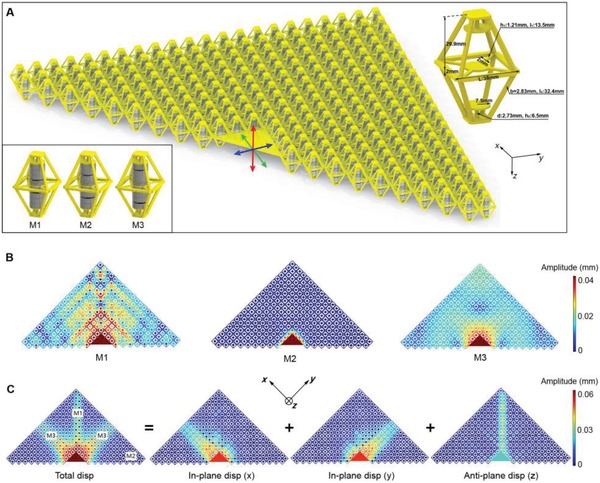
Proposed tunable locally resonant metamaterial. a) A 2D assembly with a trigonal configuration, three tunable modes shown in the inset. b) Numerical investigation of wave propagation with all cells in M1, M2, and M3. c) Three waveguides formed by the designed modes. Total displacement amplitude, and displacement amplitude of *x*, *y*, and *z* directions, where “disp” means displacement.

For convenience in expressing propagation of elastic waves, the axial direction of the electromagnets is defined as along the *z*‐axis, while the *x*‐ and *y*‐axes are parallel to the two sides of the central square of the frame. As shown in Figure 1a, a trigonal periodic lattice is assembled in the *xy* plane, and a rigid triangular plate is added at the center of the hypotenuse. A harmonic excitation at 99 Hz with *x*, *y*, and *z* vibration directions is applied to the rigid plate. The propagation of elastic waves in the lattice with all unit cells switched to M1, M2, and M3 is simulated in sequence by using the finite element method (FEM). All resulting displacements are shown in Figure 1b. We call waves vibrating in the *xy* plane “in‐plane waves,” and those vibrating along the *z*‐axis “anti‐plane waves.” Only anti‐plane waves can propagate through the lattice defined by M1. Conversely, only in‐plane waves can propagate with all unit cells tuned to M3. Switching modes to M2, neither anti‐plane waves nor in‐plane waves can propagate. Based on the selective propagation of the elastic waves in the three modes, three routes are then built. As shown in Figure 1c. M2 is set as background mode, two orthogonal routes (marked with red dots) are switched to M3 while the central route (marked with black dots) is switched to M1. Under the same excitation, the simulated results show that in‐plane waves polarized along the *x* (or *y*) direction propagate only along the *x*‐ (or *y*‐)axis route, and anti‐plane waves propagate only along the central route, thus forming three waveguides. The results indicate that, by switching modes for the unit cells, the tunable metamaterial exhibits polarizer‐like characteristic, which causes linearly polarized elastic waves to be filtered out from hybrid elastic waves.

To understand the physics underlying polarizer‐like characteristics, bandgaps of the metamaterial under the three modes are considered. According to the locally resonant effect,^24^ two kinds of independent resonators are formed in each mode at low frequency—electromagnets with the cylindrical cantilevers at the ends and electromagnets with the rectangular cantilevers at the center. Therefore, in‐plane bandgap and anti‐plane bandgap are further introduced. Band structures numerically investigated through dispersion analysis^27^ are shown in **Figure** **2**a. The in‐plane bandgap of M1 appears at frequencies ranging from 62 to 100 Hz, where only the anti‐plane vibration mode of the unit cell is observed. Switching modes to M2, the starting frequency and the bandwidth of the in‐plane bandgap is changed as a result of a variation in the mass ratio between the resonator and the substrate. With the continuous decreasing of the mass at the two ends, M3 has the highest starting frequency (151 Hz) and the narrowest bandwidth (19 Hz) for in‐plane bandgap. On the other hand, for anti‐plane bandgap caused by the central resonator, the bandgap of M1 appears in frequencies ranging from 125 to 142 Hz. When switching to M2 or M3, the mass of the resonator increases, thus the start frequency decreases and bandwidth starts to increase. In particular, in‐plane bandgap and anti‐plane bandgap of M2 overlap in the range 90–115 Hz, exhibiting a complete bandgap. Therefore, when excitation is applied at 99 Hz, the in‐plane wave can only travel through the two waveguides with M3, whereas the anti‐plane wave only travels through the central waveguide with M1. In addition, effective density has been successfully used to describe and predict wave propagation in elastic metamaterials.^28^, ^29^ Figure 2b shows the relationships between the effective density and frequency for M1, M2, and M3 under in‐plane and anti‐plane vibrations. Notably, the frequency ranges of bandgaps fall precisely into those of the effective negative density, indicating that bandgaps are caused by the two types of locally resonant systems (see details in the Supporting Information).

**Figure 2:**
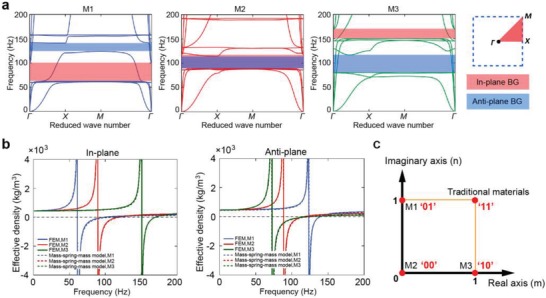
a) Band structures of M1, M2, and M3 under 2D arrangement. The in‐plane and anti‐plane bandgaps are shaded in red and blue, respectively. “BG” means bandgap. b) Effective density of the three modes under in‐plane and anti‐plane vibrations. c) Complex digital codes in complex plane.

By coding “0” and “1,” the nonpropagation and propagation characteristics of in‐plane and anti‐plane waves in the three modes can be digitalized. We propose a unique principle of complex digital codes that develops binary digits from real axes (“*z* = *m*,” *m* = 0, 1) to complex plane (“*z* = *m* +*in*,” *m*, *n* = 0, 1). Due to the similar orthogonality and independence between in‐plane and anti‐plane waves, the real and the imaginary axes can represent in‐plane and anti‐plane waves, respectively, as shown in Figure 2c. For M1, due to the nonpropagation of in‐plane waves and the propagation of anti‐plane waves, the digital code is “01” (“*m* = 0, *n* = 1”). Similarly, M2 and M3 correspond to the codes “00” and “10,” respectively. For traditional materials, both in‐plane and anti‐plane waves are allowed, and are considered to be “11.” Here the digital mode “11” can be realized by breaking the symmetry of the electromagnets' distribution in the unit cell, and the detailed feasible solution is shown in the Supporting Information. Therefore, the 2‐bit codes “00,” “01,” “10,” and “11” are formed to characterize the four scenarios. The digital description of the coding metamaterials can significantly reduce the number of steps and the complexity in designing elastic metamaterials. Also, since these digital unit cells are independently controlled, the functionality of the metamaterials can be switched in real time by simply changing the input coding sequences, which yields programmable metamaterials.^17^, ^30^, ^31^, ^32^

To validate the theoretical and numerical predictions, 1D transmittance experiments are conducted. A sample consisting of eight cells is fabricated as shown in **Figure** **3**a, and the experimentally obtained transmittance values are shown in Figure 3b,c. We also calculate the numerical transmittance curves by finite element method. The overall trend of the experimental transmittance curves is consistent with that of the calculated values. Also, the frequency ranges of in‐plane bandgaps highlighted in red and those of the anti‐plane bandgaps highlighted in blue coincide well with regions of corresponding negative transmittance. As to the experimental transmittance, the resonant peaks were erased at higher frequencies by the damping in the sample and submerged in the measurement noise^33^, ^34^ (see details in the Supporting Information). Note that the additional regions with negative anti‐plane transmittance were induced by directional bandgaps under 1D arrangement (seen in the Supporting Information).

**Figure 3:**
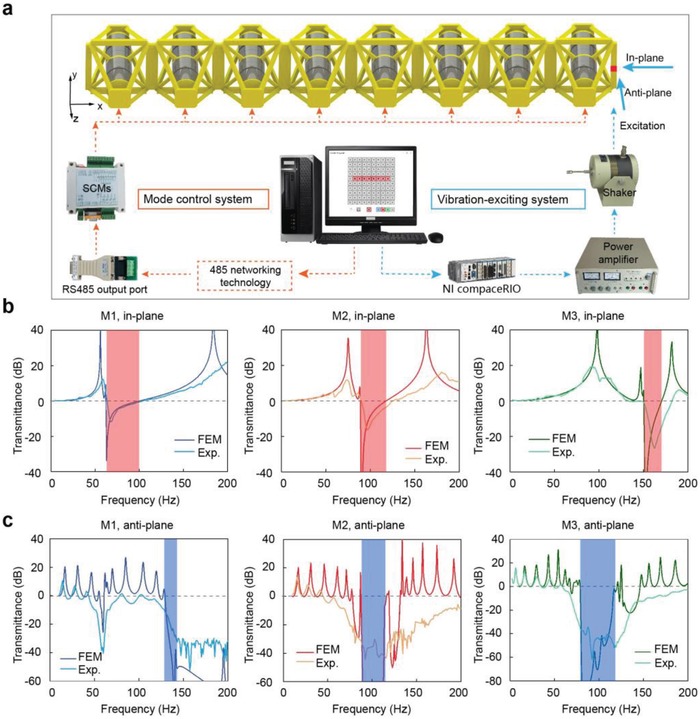
a) 1D experimental set‐ups. b,c) Numerical and experimental transmittance curves of the 1D lattice under in‐plane and anti‐plane vibration, respectively. The in‐plane and anti‐plane bandgaps are highlighted in red and blue shadow, respectively.

Not only in‐plane and anti‐plane waves are filtered out from hybrid waves, but the former with different polarizations—longitudinal and shear waves—also exhibits the phenomenon of selective wave filtering (Figure 1c). To reveal the filtering mechanism of the in‐plane polarized waves, an effective spring—mass model for the route defined by M3 is built (seen in the Supporting Information). The dispersion relation indicates that both longitudinal and shear waves are attenuated. But shear waves attenuate drastically, whose amplitude is less than one tenth of longitudinal waves at the exit of the waveguide. Therefore, only longitudinal waves show to propagate in the route, i.e., a wave vibrating along the *x* (or *y*) direction only propagates along the waveguide of this direction.

To apply the digital metamaterial possessing polarizer‐like characteristic, a 2D lattice consisting of 6 × 6 unit cells is fabricated (**Figure** **4**a). A polyethylene film with the thickness of 0.1 mm is stuck on the top of the lattice, and three stacks of black sesame are uniformly laid on the film. A harmonic excitation at 92 Hz, containing the same *y*‐ and *z*‐direction amplitude, is applied to the frame (Figure 4b, colored in red). All unit cells are first switched to M1 using the software, the experimental vibrational response at steady state shows that sesame particles are distributed in an array (Figure 4b). We also simulate the vibrational response of the lattice with a film, in which the regions corresponding to the locations of the sesame particles exhibit minimal vibration. With the code “01” of M1, anti‐plane wave propagation is dominant in the array. The anti‐plane wave further induces the intense anti‐plane vibrations of the film, resulting in the sesame particles locating in regions of lower amplitudes (orange dotted circles). When switching mode to M3, the sesame particles exhibit evolution by crawling forward instead of gathering, and are eventually scattered on the film. The code of M3 is “10,” thus in‐plane waves prevail in the lattice, which further drive the film to vibrate along the in‐plane direction. The pattern evolutions of black sesame are recorded in Videos S1 and S2 in the Supporting Information.

**Figure 4:**
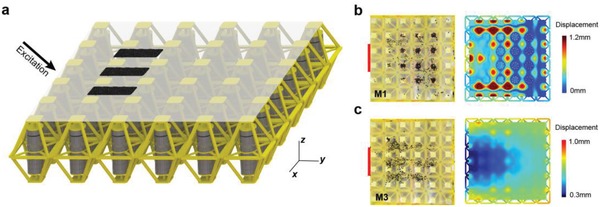
a) 3D‐printed lattice with a polyethylene film stuck on the top of the lattice, and three stacks of sesame particles deposited on the film. b,c) The experimental particles distribution and the numerical modal patterns of the lattice with a film under M1 and M3, respectively.

Compared with the past work,^17^ our proposed metamaterial possesses three tunable modes, and can be assembled into 3D structures. In **Figure** **5**b, a small square array and a big hollow array are built, joined by a rigid square sheet at the bottom. In that case, elastic waves in the two arrays can propagate independently. Under a hybrid excitation at 88 Hz, waves vibrating in *xy* plane show to travel in the external loop while waves vibrating in *z* axis travel in internal array. This feature of selective wave filtering can be applied to manipulate the vibration of devices. To simplify, one flexible slim tube is added to the top‐center of the lattice. For comparison, a similar homogeneous material is built, in which both *xy*‐plane and *z*‐axis waves prevail in the tube (Figure 5c and Video S3, Supporting Information). For the lattice structure, the amplitude ratio between longitudinal and horizontal vibrations in EM1 is 20.6, namely, longitudinal vibration is prevalent while horizontal vibration is negligible (Figure 5d and Video S4, Supporting Information). Compared to the homogeneous material, the horizontal amplitude of the tube drops from 15.4 to 0.197 mm, which is promisingly used to precisely control the axial motion of soft rods. Swapping the modes to EM2, the tube primarily performs horizontal vibrations with the amplitude ratio 0.053 between longitudinal and horizontal vibrations (Figure 5e and Video S5, Supporting Information). And the amplitude of longitudinal displacement drops from 2.59 to 0.906 mm due to the attenuating of anti‐sheet vibration in M3. EM2 can contribute to the equipment that requires to move horizontally but keeps longitudinal stability, such as shaking tables and blenders. Both flexural and longitudinal vibrations can be utilized by adding other tubes on the outside array.

**Figure 5:**
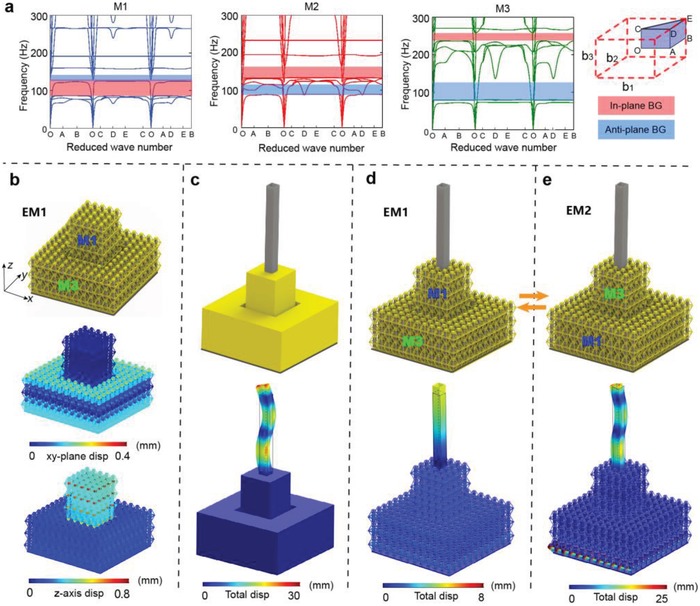
a) Band structures of M1, M2, and M3 under 3D arrangement. Structural configurations and displacements of b) 3D lattice structure of metamaterials, c) homogeneous structure with a tube, and d,e) 3D lattice structure with a tube under EM1 and EM2 mode, respectively. “disp” means displacement.

In summary, we propose an elastic metamaterial inspired by the abacus that can filter out polarized elastic waves along specific paths from hybrid waves. These unique phenomena all arise as a result of the decoupling of various vibrational modes, structural tunability and expandability. Owing to its special design with independent resonators, two kinds of locally resonant systems are formed in each mode. Thus, polarized bandgaps are created in each mode. The digital nature in each mode leads to the implementation of 2‐bit programmable metamaterials by developing complex binary digits. The superior expandability along the three directions also increases its application value as tunable lattice structure. This study provides a novel means of selective wave filtering from hybrid elastic waves, and offers promise for vibration control on particle distribution and flexible structure.

## Conflict of Interest

The authors declare no conflict of interest.

## Supporting information

Supplementary

Supplementary

Supplementary

Supplementary

Supplementary

Supplementary
