# Supplementary material for: Designing 3D Digital Metamaterial for Elastic Waves: From Elastic Wave Polarizer to Vibration Control
Source: Adv Sci (Weinh). 2019 Jun 14;6(16):1900401. doi: 10.1002/advs.201900401 (PMC6702649; doi:10.1002/advs.201900401)
Supplement: Supplementary file 1 — Supplementary [file ADVS-6-1900401-s002.pdf]

Copyright WILEY-VCH Verlag GmbH & Co. KGaA, 69469 Weinheim, Germany, 2019.

ADVANCED SCIENCE
Open Access

Supporting Information

for *Adv. Sci.,* DOI: 10.1002/advs.201900401

Designing 3D Digital Metamaterial for Elastic Waves: From Elastic Wave Polarizer to Vibration Control

Huan Liu, Quan Zhang, Kai Zhang, Gengkai Hu, and Huiling Duan**

Copyright WILEY-VCH Verlag GmbH & Co. KGaA, 69469 Weinheim, Germany, 2019.

Supporting Information

Designing 3D Digital Metamaterial for Elastic Waves: From Elastic Wave Polarizer to Vibration Control

Huan Liu[1], Quan Zhang[2], Kai Zhang[2, 3, *], Gengkai Hu[2, 3], and Huiling Duan[1, 4, *]

[1]State Key Laboratory of Turbulence and Complex Systems, Department of Mechanics and Engineering Science, BIC-ESAT, College of Engineering, Peking university, Beijing, 100871, China
[2]School of Aerospace Engineering, Beijing Institute of Technology, Beijing, 100081, China
[3]Key Laboratory of Dynamics and Control of Flight Vehicle, School of Aerospace Engineering, Beijing Institute of Technology, Beijing, 100081, China.
[4]CAPT, HEDPS and IFSA Collaborative Innovation Center of MoE, Peking university, Beijing, 100871, China

* E-mail: zhangkai@bit.edu.cn; hlduan@pku.edu.cn

Material property

Mass density and Poisson's ratio of the printing photosensitive resin (RGD836, Veroyellow) are 1170 kg m^{-3} and 0.33, respectively. In our work, the octahedral frame is printed along *z* axis (**Figure S1a**) at room temperature with 10% humidity. Since beams in different positions have different relative printing directions, Young's modulus for different beams is required to test. The two key parts, cylindrical beams on the two ends and rectangular beams in the center, are first tested by using uniaxial tensile tests (MTESTQuattroTM material testing system, ADMET Inc.) at a strain rate of 0.01 S^{-1}. As shown in **Figure S1b**, Young's modulus of upper and lower cylindrical beams is around 2 GPa, while that of the rectangular beams is around 1 GPa. The outer frame is also measured and has almost the same value as cylindrical beams. Owing to the thickness of three circular plates close to the rectangular beams, we assume Young's modulus of the three plates is 1GPa. The mass of each electromagnet is 4.2 g and the calculated density ρ_e is 4960 kg m^{-3}. Since the electromagnet is coated with steel, Young's modulus and Poisson's ratio of the electromagnet is assumed to be 185 GPa and 0.33,

respectively. In the 2D experiment shown in **Figure 4**, the polyethylene film with the thickness of 0.1mm is used. Young's modulus, density and Poisson's ratio are 1.2 GPa, 2100 kg m^{-3} and 0.33, respectively.

Structure of the proposed metamaterials

As shown in **Figure 1a**, four unrestricted electromagnets can move freely in the octahedral frame because the distance between the adjacent plates is larger than the sum of the thickness of electromagnets on one side. If no current in the electromagnets, the four unrestricted electromagnets might drop out from the unit cell when the unit cell is horizontally placed (the axis direction of the electromagnets vertical to the gravity direction). For the electromagnets (XDA-15/5), a cylindrical hole is set in the center with the radius of 1.2 mm (as shown in **Figure S2a**). We then punch another three holes in the center of the three circular plates with the radius of 0.1 mm. A thin cotton thread is used through the holes to link the electromagnets and frame together, as shown in **Figure S2b**. To prevent falling down of the electromagnets, we can also fabricate four slim and flexible bars in the octahedral frame by multi-material 3D-printed technology, as shown in **Figure S2c**. One flexible printing material—TangoPlus (FullCure 930, TangoPlus; Objet350, Stratasys, USA) can be used to fabricate these bars. The property of TangoPlus can be described by Arruda-Boyce hyperelastic model shown in Ref. [1]. The four bars have the same radius (0.5 mm) and the height (7 mm) as shown in **Figure S2c**. Because the cotton thread and bars are thin and soft enough, we prove by numerical simulation that the thread and the bars will not affect the characteristics of the metamaterial.

Intelligent mode control system

To control the modes of each cell more conveniently, an intelligent mode control system with a user friendly software is established. The simple process of the system can be described as follows: First, each unit cell in the metamaterial is connected with a small single chip

microcomputer (SCM, JMDM-4DI8DOMT, China). By using SCM, the current of the eight electromagnets can be controlled independently. Second, each SCM can receive signals from the computer. The signals SCM executing will make the unit cell switch into the desired mode. Then we adopt RS485 network technology, by which all SCMs can communicate with one computer simultaneously. Here, a serial converter (UT-2201, UTEK, China) is used to convert RS232 to RS485 for each SCM, preparing for the following network technology. After that, all the serial converters are connected together as shown in **Figure S3**. Finally, a user friendly software is designed to send instructions to all the SCMs. In the software, the three modes are expressed by the three primary colors (blue for M1, red for M2 and green for M3, respectively). 10 × 10 buttons are set in the software panel and the number of the buttons can be customized as needed. By specifying physical address for each SCM, the corresponding button can directly and independently communicate with the SCM. Particularly, when the number of unit cells is less than 100, we can just manipulate buttons that have the same physical address as the SCMs, and other buttons will not work. If the number of unit cells is more than 100, we can achieve mode control by adding the number of buttons. The schematic diagram is shown in **Figure S3**.

Experimental method on transmittance through 1D lattice structure

One-dimensional experiments are conducted to verify the calculated numerical transmittances. A lattice structure with 8 × 1 unit cells is built and suspended by strings, keeping the central plane coincident with the gravitational direction. The set-up for in-plane transmittance are shown in **Figure S4a**. An electrodynamic shaker (HEV-50, Nanjing Foneng, China) is used to apply harmonic displacement excitations. When direction of the applied excitation is coincident with the axial direction of the one-dimensional sample, in-plane vibration is induced. while the excitation direction is converted perpendicular to the central plane, anti-plane vibration is induced, as shown in **Figure S4b**. Both displacement responses are

recorded using two laser sensors (IL-30, KEYENCE, Japan), targeting the two ends of the lattice. The transmittance is computed as T=20log$_{10}$(A_1(f)/A_2(f)), where A_1 and A_2 are the amplitude of the right and left end of the lattice, respectively. Here the frequency range where the transmittance is less than zero is considered as a bandgap.

Equivalent Mass-spring-mass model for the unit cell

The physics of the unit cell can be understood by two "mass–spring–mass" models. As applying in-plane excitation, the electromagnets and beams on the two ends act as the local resonator. The equivalent "mass–spring–mass" model of in-plane vibration is shown in **Figure S5a**. Here $m_1^{(1)}$ contains the outer octahedral frame and the central electromagnets. The mass of the resonator $m_2^{(1)}$ is the mass sum of the electromagnets on the two ends, because the two identical resonators are connected in parallel to the frame. $K^{(1)}$ is the effective stiffness of the resonator. For anti-plane excitation, electromagnets and beams in the center act as the resonator for anti-plane vibration, thus the vibration of central electromagnets interacts with anti-plane wave due to the locally resonant effect. As shown in **Figure S5b**, $m_1^{(2)}$ includes the outer octahedral frame and the electromagnets on the two ends, and $m_2^{(2)}$ is the mass of the central electromagnets. $K^{(2)}$ is the effective stiffness of the central resonator. The superscripts of the parameters are the model number to distinguish the two models, '(1)' and '(2)' stands for models of in-plane vibration and anti-plane vibration, respectively. Since the two equivalent models have the uniform configuration, the effective mass of both in-plane vibration and anti-plane vibration can be estimated through a simple "mass–spring–mass" Lorentz model:

$$m_{eff} = m_1 + \frac{m_2 \omega_0^2}{\omega_0^2 - \omega^2} \tag{1}$$

where m_1 and m_2 are the masses of substrate and the resonator. ω_0 is the local resonance frequency of the resonator on the two ends, which equal to $\sqrt{K^{(1)}/m_2^{(1)}}$ for in-plane vibration and $\sqrt{K^{(2)}/m_2^{(2)}}$ for anti-plane vibration.

Wave filtering mechanism of in-plane polarized waves

In order to reveal the wave filtering mechanism of in-plane polarized waves, a two dimensional spring-mass model is further established as shown in **Figure S6a**. Because in-plane wave and anti-plane wave can't propagate in M2 (99 Hz falls into the complete bandgap of M2), it's reasonable to consider M2 as the fixed boundaries. Therefore, the problem of in-plane wave propagating in the metamaterial is transformed into the problem of in-plane wave propagating in route of M3 with fixed boundary. The stiffness between the boundary and the unit cells is given by the extensional spring K_1 and the shear spring G_1, and the stiffness between the cells along the route is given by K_2 and G_2. For convenience in expressing various in-plane polarized waves, and analogously with the behavior of a homogeneous solid, the corresponding in-plane polarized waves can be denoted as longitudinal waves and shear waves. In order to simplify calculating, the route with supercell M3 is built (see **Figure S6b**). The governing equations of in-plane wave motion in the route can be expressed as

$$\begin{aligned} m_{eff}\frac{d^2u_1^j}{dt^2}+K_2\left(2u_1^j-u_1^{j-1}-u_1^{j+1}\right)+2G_1u_1^j=0 \\ m_{eff}\frac{d^2u_2^j}{dt^2}+2K_1u_2^j+G_2\left(2u_2^j-u_2^{j-1}-u_2^{j+1}\right)=0 \end{aligned} \quad (2)$$

where u_1 and u_2 are the displacement of longitudinal waves and shear waves, respectively; index j stands for the position of supercell M3. The corresponding dispersion relations for both longitudinal and shear waves can be obtained from equation (1) expressed as

$$k_1 = \frac{1}{L}\arccos\frac{2(G_1+K_2)-m_{eff}\omega^2}{2K_2}$$
$$k_2 = \frac{1}{L}\arccos\frac{2(G_2+K_1)-m_{eff}\omega^2}{2G_2} \qquad (3)$$

according to equation (3), we can find that the selective propagation behavior of in-plane waves depends on the value of wave number k_1 and k_2 under certain frequencies, which are decided by the physical parameters (G_1, G_2, K_1 and K_2) and dynamic parameters (m_{eff}, ω). In our case, the parameters, G_1, G_2, K_1 and K_2 are 94 KPa, 390 KPa, 700 KPa and 2.2 MPa, which are obtained by numerical calculation (COMSOL Multiphsics v5.3, structural mechanics module, solid mechanics module, stationary study). m_{eff} is calculated as 0.15 Kg (COMSOL Multiphsics v5.3, structural mechanics module, solid mechanics module, Frequency domain study). ω is the circular frequency, which equals to $2\pi f$ (f = 99 Hz). Taking all the parameters into equation (3), wave number k_1 and k_2 can be obtained as $0.24i/L$ *and* $1.65i/L$, respectively. For the route with M3, the in-plane polarized wave motion can be written

$$u_1 = \left(A_1 e^{-0.24x/L}\right)\cdot e^{-i\omega t}$$
$$u_2 = \left(A_2 e^{-1.65x/L}\right)\cdot e^{-i\omega t} \qquad (4)$$

where index i is the unit of complex number and x is the spatial position. We can obtain that the remaining amplitude of longitudinal wave is approximately 10% ($|u_1/A_1| = e^{-2.4} \approx 10\%$) at exit end ($x/L$=10) of the waveguide, while that of shear wave is less than 1% ($|u_2/A_2| = e^{-16.5} < 1\%$).

To further compare the attenuation coefficient of the two types of in-plane waves, the transmittances of shear wave and longitudinal wave in one branch route with the range from 92 Hz to 100 Hz is obtained by FEM (COMSOL Multiphysics, structural mechanics module, solid mechanics module, frequency-domain study). It’s shown that more than 10% of the longitudinal wave can travel through the route. In comparison, the shear wave suffers severe

attenuation, just less than 1% can be received at the exit of the route. Namely, the shear wave can be treated as not propagating in the routes. Theoretical prediction shows a good agreement with the simulation results, indicating our theoretical model is reasonable.

Effective density of the metamaterial

In the numerical simulation (COMSOL Multiphysics 5.3v, structural mechanics module, solid mechanics module, frequency-domain study), by applying time-harmonic displacement constraints with $u = Ae^{i\omega t}$, $v = 0$, and $w = 0$ for in-plane excitation, and $u = 0$, $v = 0$, and $w = Ae^{i\omega t}$ for anti-plane excitation along the boundaries of the central square of the frame, the effective density can be expressed as:

$$\rho_{eff} = -\frac{F}{\omega^2 A V_{eff}} \tag{5}$$

where F is the amplitude of the effective resultant force on the four boundaries of the central square, which turns into F_x for in-plane vibrations and F_z for anti-plane vibrations. A and ω are the amplitude and angular frequency of the applied displacement, respectively.

In theory, based on the effective medium method, the effective density under in-plane and anti-plane vibration can be uniformly estimates through the "mass–spring–mass" Lorentz model:

$$\rho_{eff} = \frac{m_1}{V_{cell}} + \frac{m_2}{V_{cell}} \cdot \frac{\omega_0^2}{\omega_0^2 - \omega^2} \tag{6}$$

where m_1 and m_2 are the mass of substrate and resonator, respectively; ω_0 is the locally resonant frequency of the resonator that can be numerically obtained by FEM. $V_{cell} = L \times L \times H$, L and H are width and height of the frame, respectively.

Band structure investigation of the two-dimensional metamaterial

The direct lattice of the proposed metamaterial is defined by lattice vectors a_1 and a_2, as shown in **Figure S7a**. While the reciprocal lattice defined by b_1 and b_2 in **Figure S7b** describes the periodicity of the frequency-wave vector relation. Band structure of the proposed metamaterial is plotted by checking all eigenfrequencies for wave number ***k*** vectors on the perimeter of the irreducible Brillouin zone (IBZ) in the reciprocal lattice as the solid triangle ΓXM. Finite element method was employed using COMSOL Multiphysics, and free wave propagation in the infinite structure was investigated using elastodynamics on the unit cell and Bloch's theorem.

Bloch mode shapes of M1, M2, and M3

Two independent, locally resonant systems are designed—electromagnets with the cylindrical cantilevers at the ends and electromagnets with the rectangular cantilevers at the center. Therefore, in-plane bandgap and anti-plane bandgap will be induced. To demonstrate this assertion, we investigate the Bloch mode shapes and the band structures corresponding to the three modes through dispersion analysis using finite-element simulations (COMSOL Multiphysics, structural mechanics module, solid mechanics module, eigenfrequency study). The unit cell in the three modes is built separately and the geometric and material parameters can be seen in the main text. Two sets of Floquet periodic boundary conditions are applied at the two pairs oppose boundaries of the center square. **Figure S8** shows the dispersion curves and the Bloch mode shapes of the six lowest bands of M1, M2, and M3 at the high-symmetry point M, and three typical bands at point Γ of the IBZ (**Figure S7a**). Notably, all these modes at point M show strong localized vibration, with only the inner electromagnets vibrating while the outer square at the center of the frame is at rest. From **Figure S8**, we can see that the generation of in-plane and anti-plane bandgaps are closely related to the vibration of the internal vibrator. Take M1 for example, the first four localized modes are associated with the

electromagnets at the ends vibrating in the in-plane direction, resulting in the in-plane bandgap (highlighted in red). On the other hand, the fifth localized mode is associated with the electromagnets at the center in the anti-plane direction. As a result, an anti-plane bandgap is formed (highlighted in blue). The sixth and seventh localized modes are associated with the rotational vibrations of the resonator, which do not interact with the propagating waves. The three bands at point Γ show the anti-phase vibrations between the resonators and substrate, which are associated with the cut-off frequency of bandgaps.

Dispersion curves and typical Bloch mode shapes for M2 and M3 are studied shown in **Figure S8b** and **S8c**. For in-plane bandgaps, the frequency ranges of the bandgaps become narrower from M1 to M2 and M3 as a result of the decreasing in mass ratio between the resonator on the two ends and substrate. But the starting frequencies shift up. The anti-plane bandgaps become wider due to the increasing of the mass ratio, and the starting frequencies shift down. Specially, in M2, the first, second, forth, and fifth localized modes associated with in-plane vibration at the two ends and have the similar frequency as the third localized mode associated with in-plane vibration at the center, which generates a complete bandgap. When switching mode from M2 to M3, the complete bandgap disappears owing to the changing of mass distribution between the resonators and the substrate.

Directional bandgap

Due to the exist of direction bandgaps, the negative regions of anti-plane transmittance are wider than we gained from band structure in **Figure 2a**. Because of the one-dimensionality of the lattice, we need only study the band structure of ΓX segment. **Figure S9** shows the band structures of the three modes of ΓX segment. Here the in-plane mode on the two ends is colored by red, the anti-plane mode of the central part is colored in blue, and the rotate mode is colored in black. The in-plane bandgap is highlighted by red shadow and anti-plane

bandgap is highlighted by blue shadow. According to **Figure 3c**, the negative regions of anti-plane transmittances agree well with the direction bandgaps shown in **Figure S9**.

Band structure investigation under 3D periodic arrangement:

Figure S10a shows the first Brillouin zone, denoted with red dashed cuboid which defined by b_1, b_2 and b_3 under three-dimensional periodic arrangement. The irreducible Brillouin zone is highlighted in yellow, with the shape of triangular prism induced by symmetry of the unit cell. The band structures corresponding to the three modes with the 3D arrangement are studied through dispersion analysis using FEM (COMSOL Multiphysics, structural mechanics module, solid mechanics module, eigenfrequency study). The unit cell in the three modes are built separately (the geometric and material parameters can be seen in the main text). Floquet periodic boundary conditions are set at the three pairs oppose boundaries of the unit cell. **Figure S10b** shows the band structures of the three modes, which exhibit the same characteristics, polarization bandgaps and bandgap shifting, as two-dimensional band structures. Here anti-plane bandgaps under 3D arrangement agree well with the start frequencies and ranges of the 2D anti-plane bandgaps. Remarkably, owing to the connection of unit cells in *z* direction, the stiffness of the upper and lower parts has increased. Thus the start frequencies of in-plane bandgaps present obvious shift-up.

Asymmetric unit cell functioning as "traditional material"

Here we propose one type of asymmetric unit cell, as shown in **Figure S11a**. The upper and lower parts have one electromagnet and three electromagnets, respectively. As for the central part, there are three electromagnets and one electromagnet on the both sides. The band structure of this asymmetric unit cell is invested as shown in **Figure S11b**. From the band structure, there is no bandgap in the frequencies ranging from 90 Hz to 100 Hz, which can act as "11" digital mode to allow the propagation of in-plane and anti-plane waves.

Transmittance curves with the effect of damping

Fig. 3b & **c** illustrates the measured transmittance curves and numerical results. Comparing with the theoretical results, one can see two obvious differences. The bandwidth becomes wider and the resonant peaks at high frequency disappear in the experimental results. The differences are due to the effect of material damping. D. Yu and G. Wang et al investigated the flexural vibration in one-dimensional periodic structure (References [33] and [34]). They concluded that the two differences between measured transmission and theoretical results—wider bandwidth and the disappearance of resonant peaks in experiment—were caused by the structural damping, especially at higher frequencies. Here, we introduce structural damping coefficient (η) of the printing material to study the effect of structural damping on the transmittance by finite element method (COMSOL Multiphysics). As shown in **Figure S12**, we can draw the conclusion that the damping depress the resonant peaks at high frequency even with a small η. In addition, bandwidth increases with the increasing of η. When η=0.1, the theoretical and experimental results match well.

Four-quadrant diagram for 2-bit coding

In addition to express our digital metamaterial by complex plane, we also introduce four-quadrant diagram to express the four scenarios of 2-bit coding. As shown in **Fig. S13**, the horizontal axis represents the in-plane wave, and the vertical axis represents the anti-plane wave. The first quadrant, whose values of the horizontal and vertical axes are positive, can stand for “traditional materials” and our asymmetric mode, since both in-plane and anti-plane waves can be propagated. Similarly, In the second quadrant, anti-plane wave can be propagated while in-plane wave cannot, which corresponds to “M1”. Similarly, the third and fourth quadrants can characterize the features of “M2” and “M3”, respectively.

Description on magnetic field force coupling produced by the electromagnets

In our work, the effective distance of magnetic field force produced by each electromagnet is about 1.5 mm, which is far less than the distance between unit cells (38 mm), namely, there is no force coupling between unit cells. In addition, since the thickness of each unit cell is 5 mm (> 1.5 mm), it is reasonable to consider that no magnetic field force interacts between non-adjacent electromagnets. Particularly, according to the controlling method, two pairs of adjacent electromagnets, located in the upper/lower and middle parts, respectively, are not powered in each mode, so the upper/lower part does not interact with the central part. Therefore, there is no force coupling between and within unit cells due to the magnetic fields produced by the electromagnets.

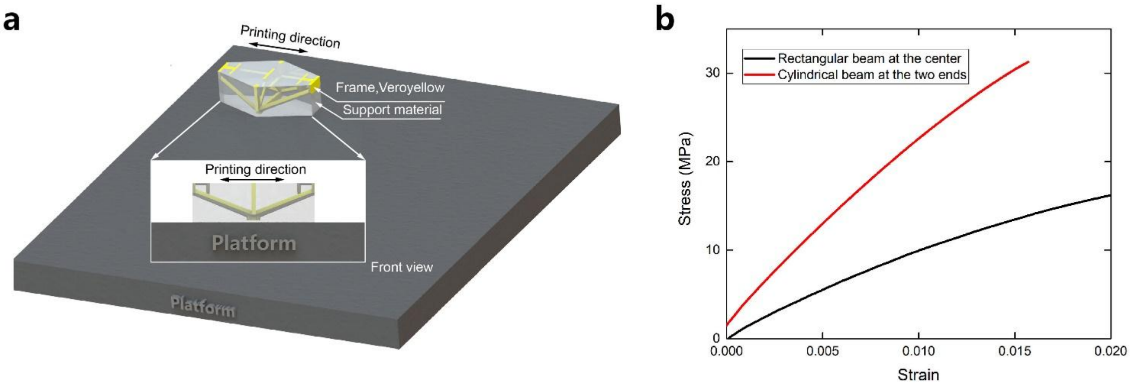

Figure S1. (a) Instruction of printing direction. (b) Stress-strain curves of the rectangular beams at the center (black line) and the cylindrical beams at the two ends (red line).

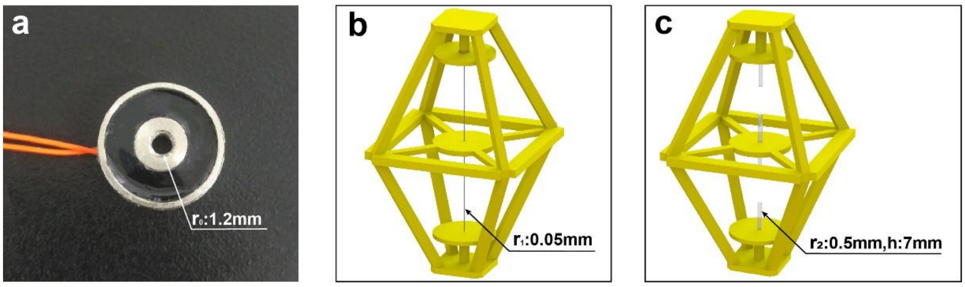

Figure S2. (a) The electromagnet. (b) The octahedral frame with a thin cotton thread. (c) The octahedral frame with four slim and flexible bars.

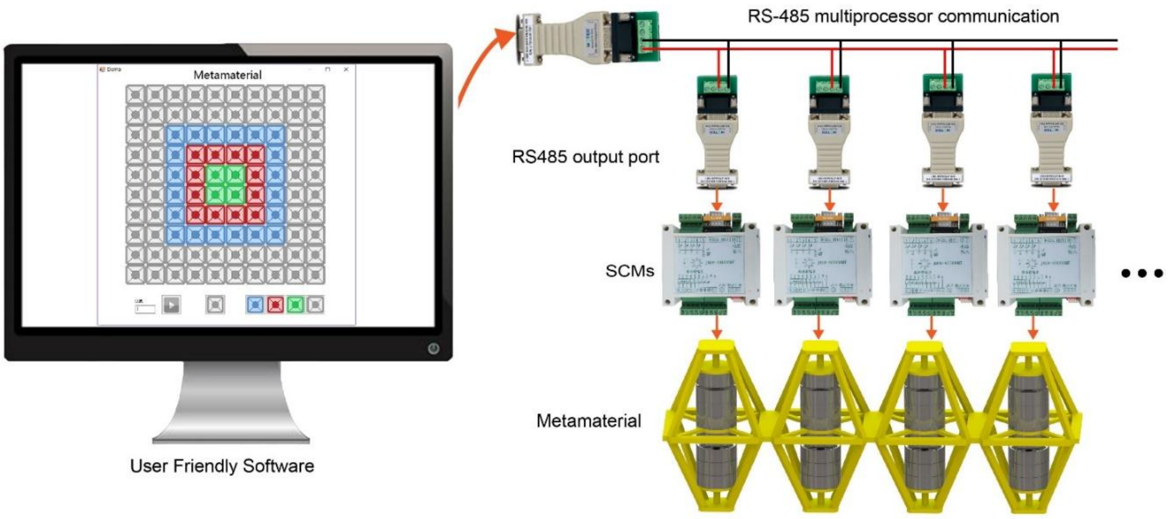

Figure S3. Intelligent mode control system.

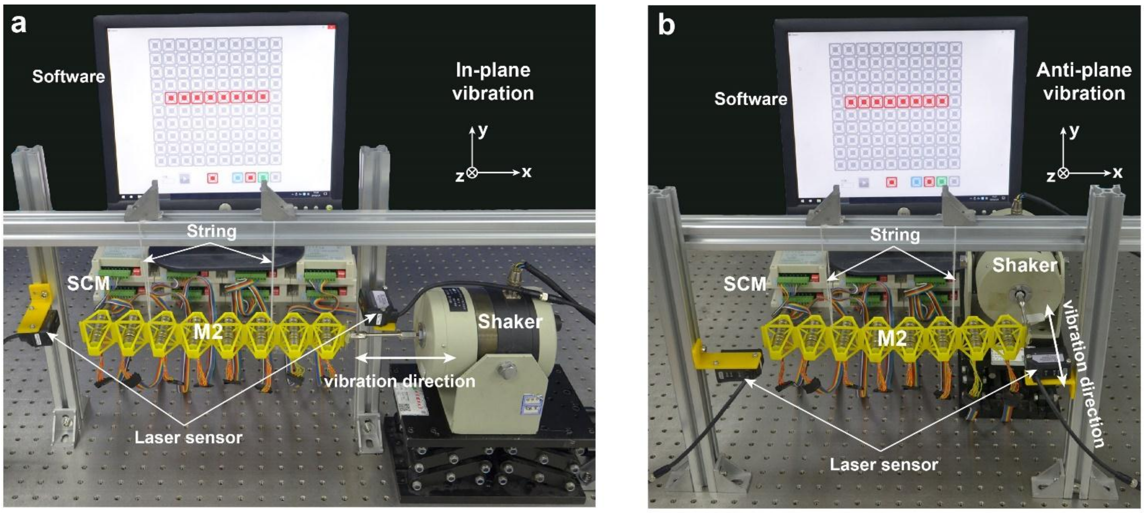

Figure S4. Experimental set-ups for one-dimensional in-plane transmittance (a) and anti-plane (b) transmittance.

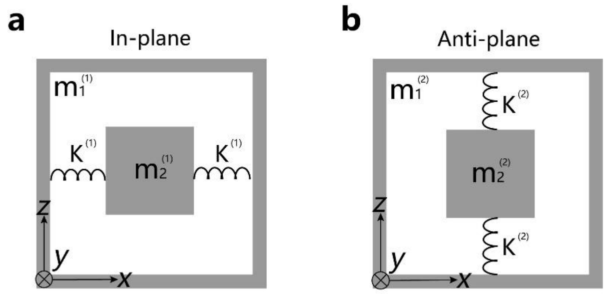

Figure S5. Equivalent mass-spring-mass model. Equivalent in-plane locally resonant system (a) for the upper and lower parts and anti-plane locally resonant system (b) for the central part.

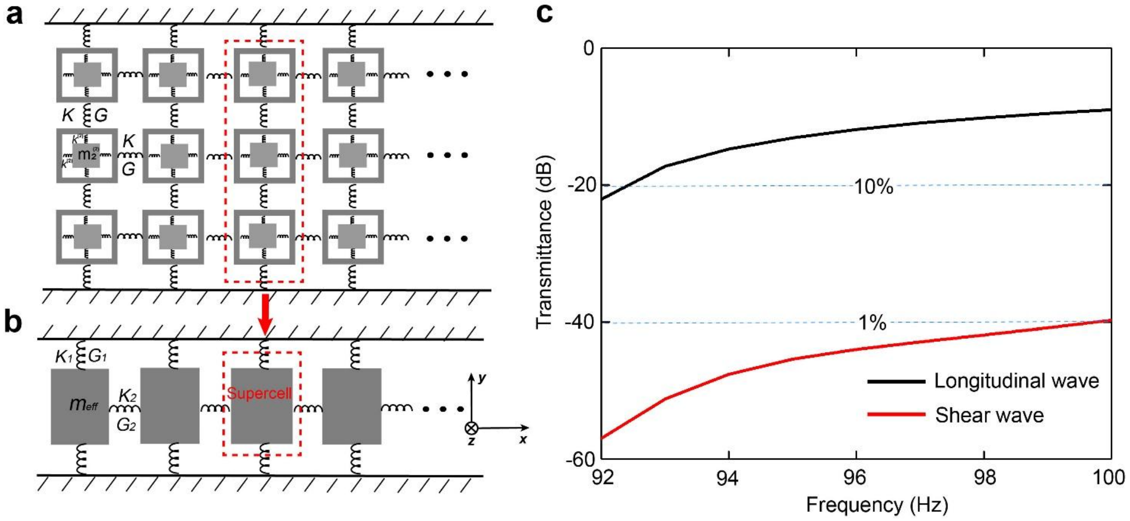

Figure S6. (a) and (b) Simplified spring-mass models. (c) Transmittance of shear wave and longitudinal wave in one branch route defined by M3 in **Figure 1c**. Two dotted lines show the positions where remaining amplitudes are 10% and 1%.

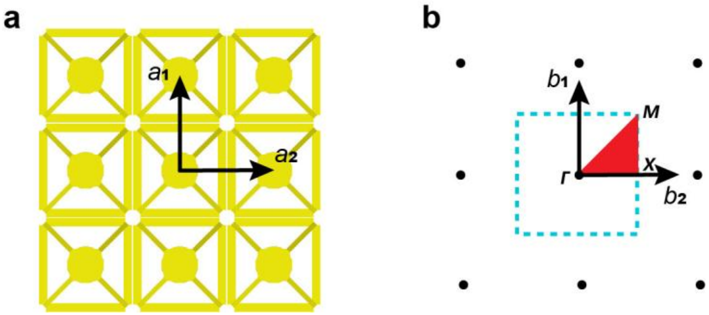

Figure S7. (a) Direct lattice defined by lattice vectors a_1 and a_2. (b) Reciprocal lattice defined by lattice vectors b_1 and b_2. The first Brillouin zone is indicated by the area inside blue rectangle, and the irreducible Brillouin zone is defined by the red area ΓXM.

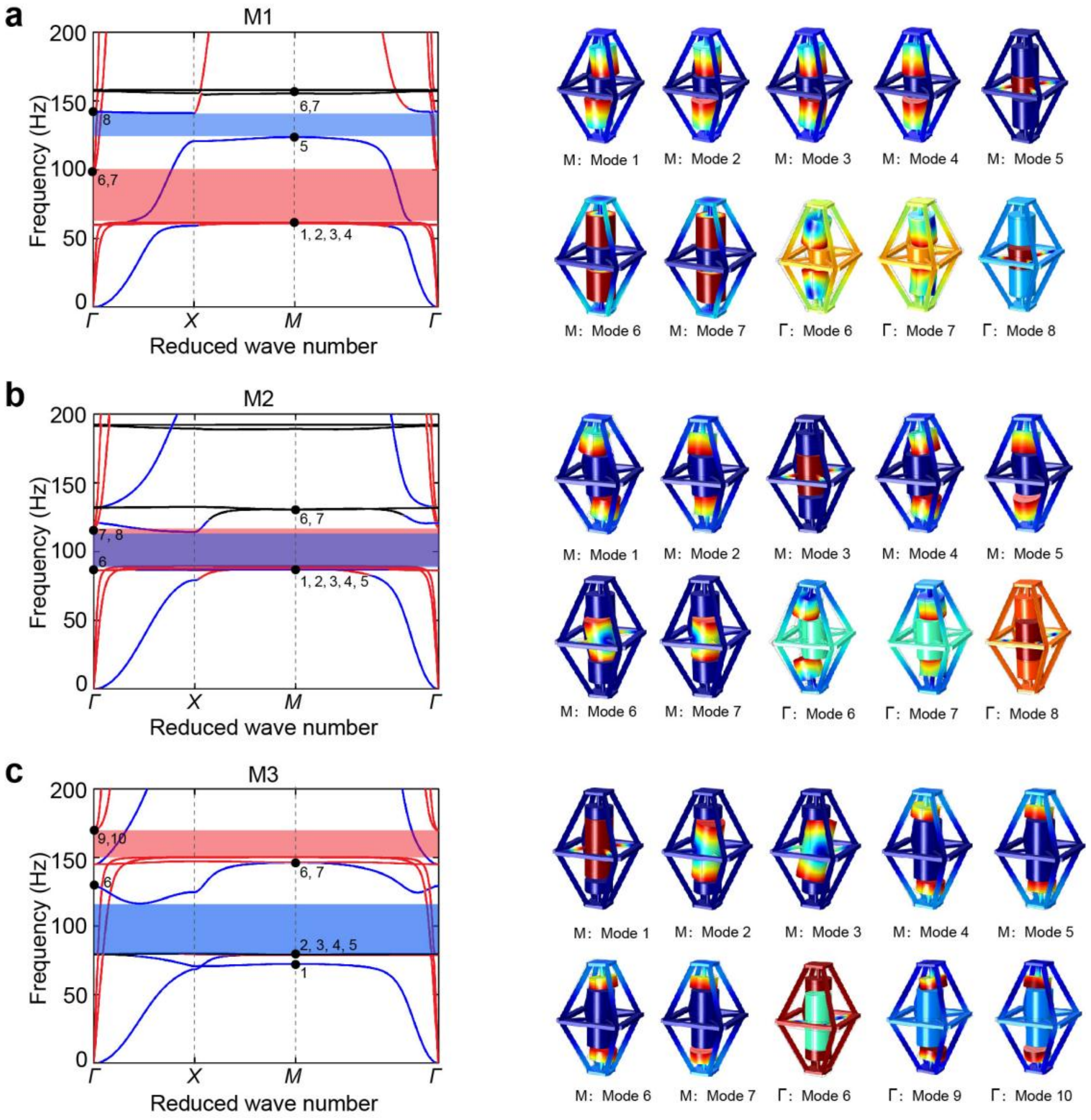

Figure. S8. Dispersion curves and typical Bloch mode shapes at the high-symmetry points *Γ* and *M* for M1 (a), M2 (b), and M3 (c). The in-plane bandgaps are highlighted in red, and anti-plane bandgaps are highlighted in blue.

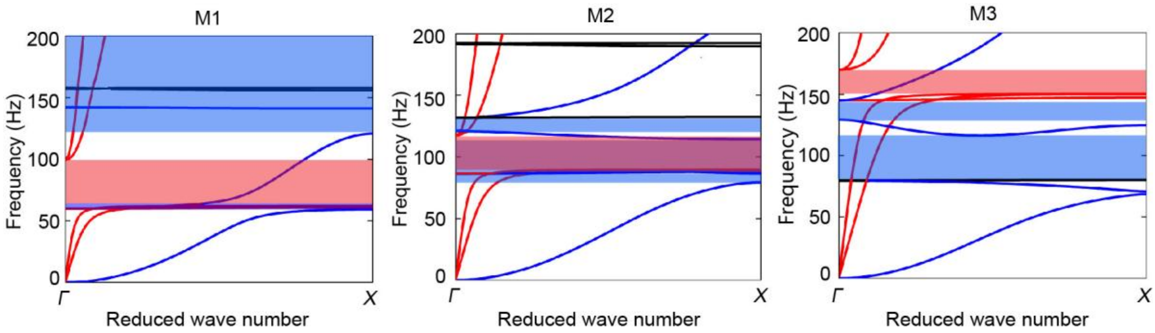

Figure S9. Anti-plane direction bandgaps of the three modes. The anti-plane bandgap highlights by blue shadow and in-plane bandgap highlights by red shadow.

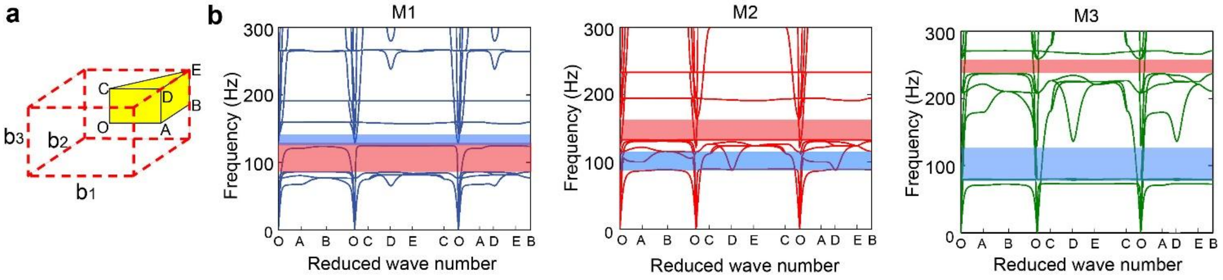

Figure S10. (a) Irreducible Brillouin zone highlighted in yellow for three-dimensional periodic lattice. (b) The band structure, where in-*xy*-plane and anti-*xy*-plane bandgaps of the three modes are shaded in red and blue, respectively.

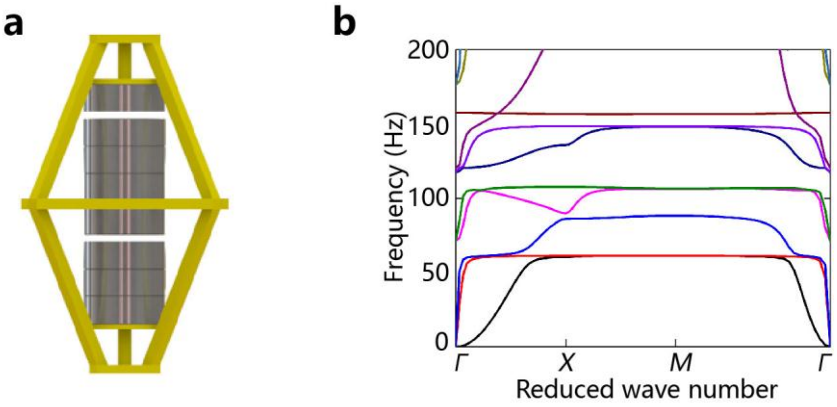

Figure S11. (a) one type of asymmetric unit cell. (b) the band structure of (a).

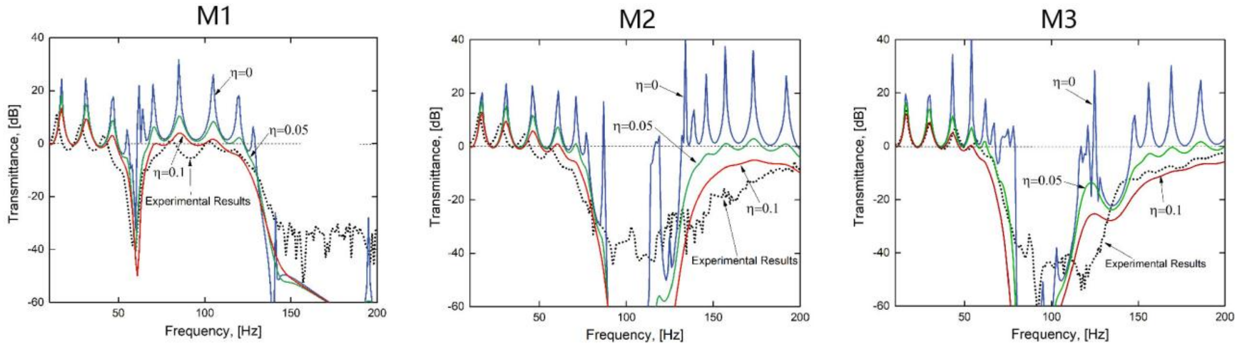

Figure S12. Calculated and measured transmittance of M1, M2, and M3. The blue, green, and red lines represent the structural damping coefficient η=0, 0.05, and 0.1, respectively. The dotted line represents the experimental results.

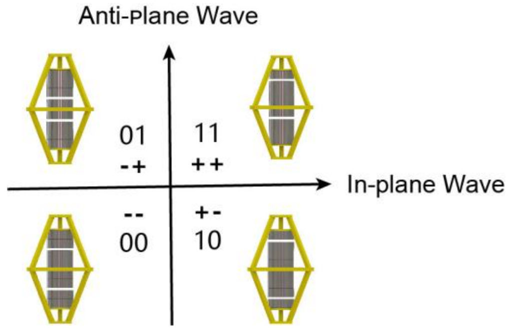

Figure S13. Four-quadrant diagram for 2-bit coding. "+" and "–" represent the "propagable" and "non-propagable", respectively.

Reference

[1] L. Wang, J. Lau, E. L. Thomas, M. C. Boyce, *Adv. Mater.* **2011**, *23*, 1524.
